# Large-Scale Screening of HCMV-Seropositive Blood Donors Indicates that HCMV Effectively Escapes from Antibodies by Cell-Associated Spread

**DOI:** 10.3390/v10090500

**Published:** 2018-09-14

**Authors:** Jessica Julia Falk, Martina Winkelmann, Kerstin Laib Sampaio, Caroline Paal, Hubert Schrezenmeier, Mira Alt, Richard Stanton, Adalbert Krawczyk, Ramin Lotfi, Christian Sinzger

**Affiliations:** 1 Institute for Virology, Ulm University Medical Center, 89081 Ulm, Germany; jessica.falk@uni-ulm.de (J.J.F.); kerstin.laib@uni-ulm.de (K.L.S.); caroline.paal@t-online.de (C.P.); 2 Institute for Transfusion Medicine, Ulm University Medical Center, 89081 Ulm, Germany; m.winkelmann@blutspende.de (M.W.); h.schrezenmeier@blutspende.de (H.S.); r.lotfi@blutspende.de (R.L.); 3 Institute for Clinical Transfusion Medicine and Immunogenetics Ulm, German Red Cross Blood Transfusion Service Baden-Württemberg–Hessen and Ulm University Medical Center, 89081 Ulm, Germany; 4 Institute for Virology, University Hospital Essen, University of Duisburg-Essen, 45147 Essen, Germany; Mira.Alt@uk-essen.de (M.A.); Adalbert.Krawczyk@uk-essen.de (A.K.); 5 Division of Infection and Immunity, Cardiff University School of Medicine, Cardiff CF14 4XN, UK; richstanton@gmail.com

**Keywords:** cytomegalovirus, antibodies, cell-associated spread, immune evasion

## Abstract

Immunoglobulins are only moderately effective for the treatment of human cytomegalovirus (HCMV) infections, possibly due to ineffectiveness against cell-associated virus spread. To overcome this limitation, we aimed to identify individuals with exceptional antibodies in their plasma that can efficiently block the cell-associated spread of HCMV. A Gaussia luciferase-secreting mutant of the cell-associated HCMV strain Merlin was generated, and luciferase activity evaluated as a readout for the extent of cell-associated focal spread. This reporter virus-based assay was then applied to screen plasma samples from 8400 HCMV-seropositive individuals for their inhibitory effect, including direct-acting antiviral drugs as positive controls. None of the plasmas reduced virus spread to the level of these controls. Even the top-scoring samples that partially reduced luciferase activity in the screening assay failed to inhibit focal growth when reevaluated with a more accurate, immunofluorescence-based assay. Selected sera with high neutralizing capacity against free viruses were analyzed separately, and none of them prevented the focal spread of three recent clinical HCMV isolates nor reduced the number of particles transmitted, as demonstrated with a fluorescent Merlin mutant. We concluded that donors with cell-to-cell-spread-inhibiting plasma are nonexistent or extremely rare, emphasizing cell-associated spread as a highly efficient immune escape mechanism of HCMV.

## 1. Introduction

Human cytomegalovirus (HCMV) is a herpesvirus that is ubiquitously distributed in the human population. Whereas primary infection in immunocompetent individuals usually causes only mild symptoms or mononucleosis-like disease, immunocompromised individuals (e.g., AIDS patients or transplant recipients) can suffer from severe disease following HCMV primary infection or reactivation. Solid-organ or hematopoietic-stem-cell transplant recipients are at high risk of HCMV infection, which can entail organ manifestations, but also indirect complications, including graft failure, graft-versus-host disease, and increased susceptibility to other infections [1,2,3,4]. Due to intrauterine infection, HCMV is also a cause of disabilities in children, often manifested as hearing loss or other neurological impairments [5]. The incidence of congenital HCMV infections varies from 0.6–0.7% in industrialized countries to 1–5% in developing countries [6], and leads to clinical findings at birth in 10–15% of cases [7,8].

Effective direct-acting polymerase inhibitors (i.e., ganciclovir, foscarnet, and cidofovir) are available, but their administration is limited due to myelotoxic or nephrotoxic side effects and the development of resistance [9,10,11,12]. Recently, a terminase inhibitor (letermovir) has been approved that appears to be less toxic, but resistance can occur [13]. For this reason, passive immunization is still a desirable alternative. Reports on the efficacy of HCMV hyperimmunoglobulins in pregnant women with primary HCMV infection [14,15,16,17] and in transplant recipients [18,19,20] are controversial. Interestingly, a combination of two neutralizing anti-HCMV antibodies was recently reported to reduce the frequency of HCMV viremia [21,22], and vaccination with recombinant glycoprotein B of HCMV provided partial protection that was correlated with antibody responses, although the mode of action of protective antibodies remains unclear [23,24,25].

The difficulties of establishing a highly effective antibody-based treatment regimen for HCMV infections may in part be due to variations in antibody sensitivity depending on the transmission mode, target-cell type, and HCMV strain. Recent clinical isolates are typically restricted to cell-associated spread in cell culture [26,27]. Subsequent passaging of clinical isolates in fibroblast cultures after isolation rapidly selects for mutations in two viral gene regions (RL13 and UL128L) that lead to the release of cell-free infectivity and restricted cell tropism [28,29]. Using passaged strains, anti-HCMV antibodies can partially reduce virus spread in endo- or epithelial cells, but are less effective against cell-associated spread in fibroblasts [30,31,32,33,34,35]. However when the cell-associated phenotype of clinical strains was recapitulated in vitro, virus spread was almost completely resistant to neutralizing antibodies; minor inhibition was only observed at very high antibody concentrations, and, as with passaged strains, it was more apparent against the virus in epithelial cells than in fibroblasts [36].

In contrast to cell-associated spread, cell-free virus can be neutralized by serum or plasma from HCMV-seropositive individuals. However, neutralization capacities vary greatly between plasma donors, and efficiency again depends on target-cell type, whereby, commonly, the infection of endo- or epithelial cells can be more effectively neutralized when compared with fibroblasts [37,38]. The exact role of various spreading modes and target-cell types in vivo is not known. Both transmission modes, however, appear to contribute in certain situations, as infectivity is almost exclusively cell-associated in the circulation [39,40,41], but often cell-free in body excretions like breast milk or urine [42,43]. It is tempting to assume that antibody preparations that can not only prevent cell-free infection of endo- and epithelial cells, but are also highly effective against cell-associated focal spread in stromal cells, could substantially improve antibody-based strategies against HCMV.

In the field of neutralizing antibodies, the identification of elite donors with exceptionally high and broad neutralization capacities has yielded significant improvement of antibody preparations [44,45,46,47]. We were interested to test whether this concept can also be transferred to identify blood donors whose plasma can, as an exception to the rule, effectively inhibit the cell-associated spread of HCMV. Therefore, we generated a reporter virus that allowed high throughput screening for cell-to-cell spread-inhibiting antibodies, and applied it to a large human blood-donor population for which neutralization capacities against cell-free HCMV infection were already known from a previous study [37].

## 2. Materials and Methods

### 2.1. Cell, Plasma, Serum, and HCMV Clinical Isolates

For propagation, human foreskin fibroblasts (HFF) were kept in “growth medium” containing minimal essential medium with 5% fetal bovine serum (PAN Biotech, Aidenbach, Germany), GlutaMAX (Life Technologies, Carlsbad, CA, USA), 100 μg/mL gentamicin, and basic fibroblast growth factor (bFGF; Life Technologies, 0.5 ng/mL). Human fetal foreskin fibroblasts (HFFF-tet) cells were immortalized with hTERT and expressed the Tet-repressor [28]. For propagation, HFFF-tet cells were cultured in “growth medium”. During experiments, both HFFs and HFFF-tet cells were kept in “growth medium” without bFGF (MEM5). Conditionally immortalized human endothelial cells (HEC-LTTs) [48,49] were cultured in endothelial cell-growth medium (Endothelial Cell Growth Medium Kit, PromoCell, Heidelberg, Germany) supplemented with 2 µg/mL doxycycline (AppliChem, Darmstadt, Germany). During infection experiments, heparin and doxycycline were omitted from the medium. Cell-culture microplates were coated with 0.1% gelatin (Sigma-Aldrich, St. Louis, MO, USA) prior to seeding of cells.

The German Red Cross Blood Transfusion Service, Baden-Württemberg-Hessen, provided ethylenediaminetetraacetic acid (EDTA)-plasma samples from their HCMV-seropositive blood donors. The Ethical Committee of the University of Ulm proved the project design resulting in vote No. 53/14. Initial tests were performed on plasma samples routinely available from blood donors. Plasma had to be recalcified prior to use in the screening because clotting factors within plasma interfered with exact pipetting. Recalcification was performed by a tenfold dilution of plasma in MEM5, followed by incubation at 37 °C for at least 2 h. Plasma samples were then stored at 4 °C until the day of the experiment. Individuals identified as highly and broadly neutralizing (“elite”) donors [37] were asked to donate serum samples.

Cell-free virus preparations of HCMV strain TB40-BAC4 [50] were used to test the neutralization capacity of sera. Recent clinical HCMV isolates were provided by the diagnostic laboratory of the Institute of Virology in Ulm. They originated from routine testing of throat swabs from patients of the Ulm University Medical Center. Sample material was applied to HFFs, and HCMV-positive cultures were then incubated for several weeks until they showed at least 50% cytopathic effect. Infected cells were then aliquoted and frozen at −80 °C, and the cell association of the HCMV isolates was tested by transferring culture supernatants onto adherent HFFs and immunofluorescence staining for viral immediate-early (IE) antigens one day after inoculation. Isolates were only used further if they were negative in this assay.

### 2.2. Generation of a Luciferase Reporter Virus Based on HCMV Strain Merlin

A Gaussia luciferase (GLuc) expression cassette was inserted in strain Merlin-pAL1502, downstream of the US34A gene. Merlin-pAL1502 is a bacterial artificial chromosome (BAC)-cloned HCMV strain that is a derivative of the repaired Merlin-BAC pAL1128 [28,51]. It has tet-operator sequences in front of the RL13 and UL128 genes. The Gaussia luciferase expression cassette consists of the HCMV immediate-early promoter, the Gaussia luciferase open reading frame, and a BGH (bovine growth hormone) polyA signal.

To insert the US34A flanking sequences, a PCR (polymerase chain reaction) product was amplified from the universal transfer construct pDrive-(IE-GLuc-PolyA)-Kan [52] with primers: 5′-CATATTATGCGTGTCCTGGTTTTTCATTTTTTGGATGTATTTGTCGCATAAAAGGCGGTGATTAATAGTAATCAATTACG-3′ and 5′-GACACGGGTTTTGTTAGGATAACAAAACTGCGTATCTGGATATATTTCATCCCCACATCCGATGCAATTTCCTCATTTTA-3′ and electroporated into the recombination-activated *escherichia coli* (*E. coli*) strain GS1783 [53] harboring Merlin-pAL1502. Following kanamycin selection, the marker cassette was removed from the BAC by an intrabacterial I-SceI digest and a Red-mediated recombination step. The integrity of the recombinant Merlin-pAL1502-GLuc was verified by restriction fragment length analysis (RFLA) and sequencing of the recombined regions. Sequence analysis revealed a nucleotide substitution (G instead of T) upstream of the luciferase-coding sequence, which did not disable expression of a functional luciferase. To obtain infectious virus particles, BAC DNA was isolated using the NucleoBond Xtra Midi kit (Macherey-Nagel, Düren, Germany) and transfected into HFFs by a calcium phosphate-based method (MBS Transfection Kit, Agilent, Waldbronn, Germany). Since producer cell cultures contain high levels of luciferase that might interfere with downstream experiments, reporter-virus preparations were purified as previously described [52].

### 2.3. Generation of Dual-Labeled HCMV Strain Merlin-pAL1502-UL32EGFP-UL100mCherry

In a first step, the EGFP template cassette was amplified from plasmid pEP-EGFPin (Addgene plasmid #60961, Cambridge, MA, USA) with primers 5′-CCGTGCAGAACATCCTCCAAAAGATCGAGAAGATTAAGAAAACGGAGGAAATGGTGAGCAAGGGCGAGGAGCT-3′ and 5′-CACTATCCGATGATTTCATTAAAAAGTACGTCTGCGTGTGTGTTTCTTAATTACTTGTACAGCTCGTCCATGC-3′ and inserted into Merlin-pAL1502 by seamless mutagenesis as described above. After removal of the selection marker, an mCherry-containing recombination fragment was generated from plasmid pEP-mCherry-in [33] with primers 5′-CCCTGCGTCTACTATCACGTCGTGGACTTTGAAAGGCTCAACATGTCGGCCTACAACGTAgtgagcaagggcgaggagga-3′ and 5′-CACGGCGTAGCACACCAGCTGCACCGAGTCTAAGAAAAGCATAGGCGTGTGCAGGTGCATcttgtacagctcgtccatgc-3′ and introduced into the genome by a second round of mutagenesis. The final Merlin-pAL1502-UL32EGFP-UL100mCherry genome was verified by RFLA and sequencing, which revealed a single amino acid deletion (R54) in the UL100 coding sequence as compared to the parental strain due to mCherry insertion. Infectious virus was generated by transfection into HFFF-tet cells as described above.

### 2.4. Detection of Viral Antigen via Immunofluorescence

Following fixation with 80% acetone, the cells were incubated with antibody E13 (Argene Biosoft, Verniolle, France) to detect IE antigens 1 and 2 (pUL122/123) [54]. Cy3-conjugated goat polyclonal antimouse F(ab’)_2_ antibody (Jackson ImmunoResearch, West Grove, PA, USA) was used as a secondary antibody. The nuclei of cells were counterstained with DAPI (4′,6-diamidino-2-phenylindole, Sigma-Aldrich). Visualization was done by fluorescence microscopy with an Axio Observer D1 microscope (Zeiss, Oberkochen, Germany).

### 2.5. Gaussia Luciferase-Based Screening of Plasma Samples for Inhibition of Cell-Associated Spread

HFFs were seeded on 96-well plates (Cellstar, Greiner Bio-One, Frickenhausen, Germany) at a density of 1.5 × 10^4^ cells per well. Two days after seeding, cultures were infected with Merlin-pAL1502-GLuc using 0.002 focus-forming units (FFU) per cell. One day after infection (1 d p.i.), supernatants were replaced with recalcified plasma samples at a 50-fold dilution in MEM5. The direct-acting antivirals ganciclovir (Cymeven, 800 µM, Roche, Basel, Switzerland) and BDCRB (2-Bromo-5,6-dichloro-1-β-d-ribofuranosyl benzimidazole, kindly provided by Jens von Einem, 0.04 mM) were included as positive controls. From previous experiments, we knew that common seropositive plasma samples do not block the cell-associated spread of HCMV and resemble seronegative plasma in this assay. Therefore, we did not include seronegative controls, but rather searched for outstanding plasma from seropositive donors that would significantly deviate from the majority of the others. After 4 days (i.e., 5 d p.i.) supernatants were replaced with fresh MEM5. After further overnight incubation (i.e., 6 d p.i.), the GLuc activity in 20 µL supernatant of each well was measured as relative light units (RLU) in a multilabel microplate reader (Hidex Chameleon, Turku, Finland). As the intention of this screening was to identify plasma samples with outstanding inhibitory capacity, RLU values of individual samples were compared with the geometric mean of all plasma samples on the plate and reported as “relative luciferase activities” (RLU_sample_/RLU_mean_). Plates were only evaluated if direct-acting antivirals had relative luciferase activities below 0.3. Plasma samples that repeatedly failed these controls were excluded. Those plasma samples showing the strongest reduction of GLuc activity were considered for evaluation via immunofluorescence staining of viral antigens.

### 2.6. Focus-Expansion Assays with Clinical HCMV Isolates

Frozen stocks of HFFs, which were infected with clinical HCMV isolates and stored at low passages, were thawed, washed, and cocultured with an excess of uninfected HFFs or HEC-LTTs in 96-well microplates (Cellstar, Greiner Bio-One). This resulted in 50–100 infected cells per well surrounded by 15,000 uninfected indicator cells. Directly after seeding, highly neutralizing sera from previously identified “elite” donors [37] were added to the coculture at a final dilution of 1/50. The direct-acting antiviral ganciclovir (GCV, 800 µM) or serum from an HCMV-seronegative donor (dilution 1/50) was used as positive or negative control, respectively. After 6 days of cocultivation, cells were fixed with 80% acetone, and IE antigen was detected via immunofluorescence staining. It is noteworthy that plasma samples repeatedly had cytotoxic effects on HEC-LTT cultures during the 6 days of incubation, and, therefore, we had to use serum instead of plasma.

To confirm the capacity of the tested sera to inhibit cell-free virus particles, HFFs were seeded on 96-well microplates (Cellstar, Greiner Bio-One) at a density of 1.5 × 10^4^ cells per well. The next day, aliquots of HCMV-TB40/E-BAC4 were preincubated with 1/50 diluted sera for 2 h at 37 °C, and the mixture was added to the HFF cultures for overnight incubation at 37 °C. Cells were then fixed with 80% acetone, and IE antigen was detected via immunofluorescence staining.

### 2.7. Analysis of Single-Particle Transmission in Cell-Associated Spread

HFFs were seeded on a 96-well microplate (Cellstar, Greiner Bio-One) at a density of 1.5 × 10^4^ cells per well. The next day, cells were infected with Merlin-pAL1502-UL32EGFP-UL100mCherry at a very low infection multiplicity (MOI), resulting in around 0.2% infected cells per well. One day p.i., supernatants were removed and plasma added to the infected cells at a 50-fold dilution. After 5 d p.i., cells were fixed with 4% paraformaldehyde (Sigma-Aldrich) in PBS for 30 min and permeabilized with a permeabilization solution (PBS with 10% sucrose (Sigma-Aldrich), 1% fetal bovine serum (PAN Biotech), and 0.5% Nonidet P40 (Sigma-Aldrich)) for 10 min, both at ambient temperature. The red fluorescent mCherry signal was enhanced by additional staining with a rabbit anti-dsRed polyclonal antibody (Clontech, Kusatsu, Japan), and Cy3-conjugated goat anti-rabbit IgG F(ab’)_2_ (Jackson ImmunoResearch). Nuclei were counterstained with DAPI (Sigma-Aldrich). Images were taken with a fluorescence microscope (Axioobserver D1; Zeiss) at a 640-fold magnification.

## 3. Results

### 3.1. Generation of Reporter Virus for High-Throughput Analysis of Cell-Associated Spread

#### 3.1.1. Cloning of Reporter Virus Merlin-pAL1502-GLuc

For the generation of a reporter virus that would facilitate the screening of a blood-donor population for cell-to-cell spread-inhibiting antibodies, we used the genetic backbone of Merlin-BAC pAL1502 [28]. This Merlin derivative contained tet-operator sequences in front of the RL13 and UL128 genes, which are known to restrict the cell-free spread of HCMV. In HFFF-tet cells harboring the tet repressor, RL13 and UL128 are conditionally repressed, which allows release of cell-free infectivity into the supernatant and prevents the selection of mutants in these genes. In primary human cells lacking the tet repressor, RL13 and UL128 are expressed, and the virus grows in a strictly cell-associated manner. Using seamless mutagenesis, a GLuc expression cassette was inserted into the Merlin-pAL1502-BAC, downstream of the US34A gene. This insertion site was chosen to minimize the risk of interference with other open reading frames as it has been reported to have very low transcriptional activity [55]. Like the parental virus, this mutant grew in a cell-free mode in HFFF-tet cells after transfection of the recombined BAC genomes. In contrast, when primary cells were infected with such cell-free virus stocks, the virus then grew in a strictly cell-associated focal manner.

#### 3.1.2. Correlation between GLuc Activity and Focal Growth

**Figure 1:**
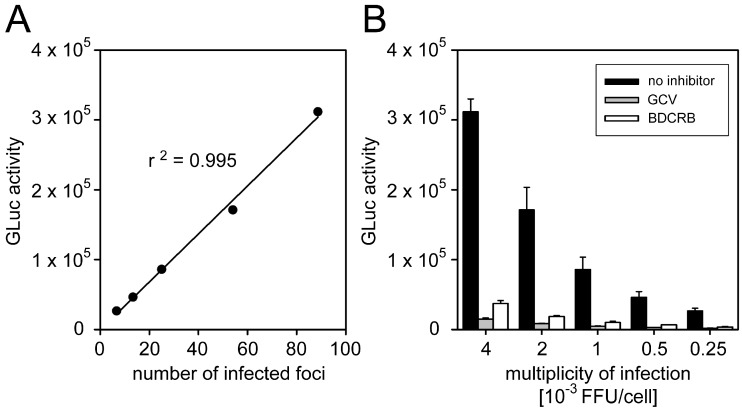
Inhibition of focal growth reflected by Gaussia luciferase (GLuc) activity. Human foreskin fibroblasts (HFFs) were seeded and infected with Merlin-pAL1502-GLuc in a dilution series in the range of 0.00025–0.004 focus-forming units/cell (FFU/cell) resulting in six to 80 infected foci per well. The following day, infected cell cultures were treated with either human cytomegalovirus (HCMV)-seronegative human plasma, or two direct-acting antivirals ganciclovir (GCV) or 2-Bromo-5,6-dichloro-1-β-d-ribofuranosyl benzimidazole (BDCRB). Six days after infection, viral immediate-early (IE) antigens were detected by immunofluorescence staining to determine the number of infected foci per well. Additionally, the GLuc activity of cell-culture supernatants was measured. (**A**) Correlation between immunofluorescence-based readout and luciferase readout. (**B**) Detection of reduced focal growth in antiviral-treated cultures by the luciferase readout at various infection multiplicities. Bars represent the mean values of triplicates and error bars indicate the standard error of the mean (SEM).

To test whether GLuc activity correlates with the extent of focal growth, HFFs (2.0 × 10^4^ cells per well) were infected with the Merlin-pAL1502-GLuc in serial dilutions at MOIs of 0.00025–0.004 FFU/cell, and viral spreading was allowed for a total of six days. At 1 d p.i., either HCMV-seronegative human plasma was added at a 50-fold dilution for uninhibited growth, or direct-acting antivirals ganciclovir (800 µM) or BDCRB (0.04 mM) were added as inhibitors of focal growth. After four days (i.e., 5 d p.i.), supernatants were replaced with MEM5 and further incubated overnight. At 6 d p.i., GLuc activities were measured in the supernatants, whereas the cells were fixed, stained for IE antigens by immunofluorescence, and analyzed regarding the numbers of foci per well (Figure 1). As expected, increasing focus numbers in the IE-Ag staining correlated with increasing Gaussia luciferase signals in a linear relationship (Figure 1A), and inhibition of focal growth by the direct-acting antivirals was reliably detected by greatly decreased GLuc activity as compared to the uninhibited control (Figure 1B). Interestingly, nucleoside analog ganciclovir, which inhibits genome replication, repeatedly yielded lower GLuc activities than terminase inhibitor BDCRB, which targets packaging of viral genomes, indicating that the luciferase readout can stably report even minor differences. At the lowest concentration of the reporter virus, six foci per well were present, as determined by immunofluorescence staining, and this low focus number was still sufficient to provide a reliable luciferase signal. In conclusion, these experiments proved that the Merlin-pAL1502-GLuc assay is reliable and sensitive enough to screen potential inhibitors regarding their effect on cell-associated focal spread of HCMV.

### 3.2. No Cell-To-Cell Spread Inhibitors Identified in Large Cohort of HCMV-Seropositive Individuals

Plasma samples of 8400 HCMV-seropositive blood donors, which we had previously characterized regarding their neutralizing effect on cell-free viruses [37], were now screened with the GLuc-based reporter assay concerning their capacities to inhibit cell-to-cell spread. Direct-acting antiviral agents and HCMV-seronegative plasma were included as positive and negative controls, respectively. Fibroblasts, seeded two days before, were infected with Merlin-pAL1502-GLuc at very low MOIs (0.002 FFU/cell). Plasma samples and control agents were added 1 d p.i., and incubated with the infected cells for four days. Cultures were then washed, further incubated overnight, and supernatants were harvested at 6 d p.i. for analysis of their relative luciferase activities. A total of 8034 samples passed the controls and could be evaluated.

**Figure 2:**
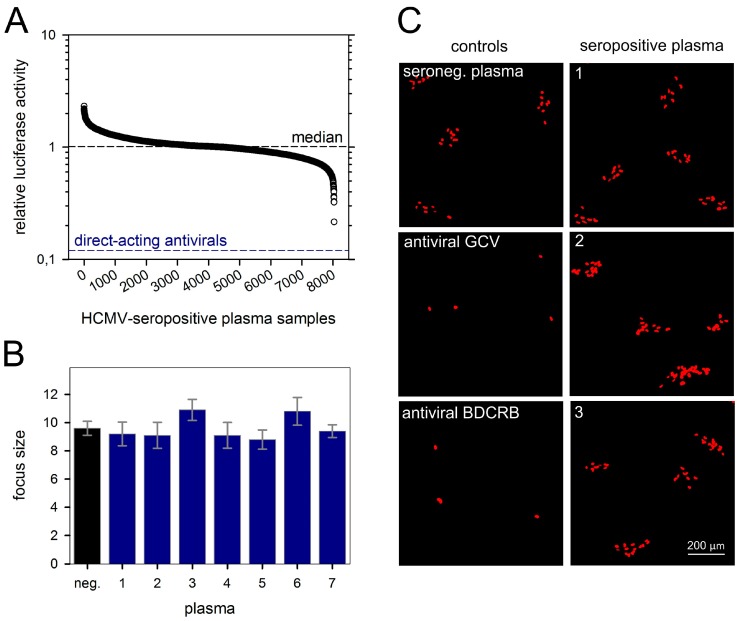
Plasma from HCMV-seropositive donors does not inhibit cell-associated viral spread. HFFs were seeded on microplates and infected with Merlin-pAL1502-GLuc at infection multiplicities of 0.002 FFU/cell. The day after infection (p.i.), plasma samples were added at a 50-fold dilution, and cells were incubated further. On day 5 p.i., medium was exchanged, and on day 6 p.i., supernatants were harvested for analysis of luciferase activities. Two direct-acting antivirals (GCV and BDCRB) served as positive controls for inhibition of cell-associated HCMV spread. (**A**) GLuc activities of individual plasma samples divided by the mean value of whole plate level (i.e., relative luciferase activity). Each circle represents plasma from one of 8034 HCMV-seropositive donors. The median of relative GLuc activities of all tested samples is depicted by a dashed horizontal line and represents unrestricted focal growth. The lower dashed line reflects maximal reduction of focal growth by direct-acting antivirals. (**B**) Among the 14 plasma samples that showed the highest reduction of GLuc activity, seven samples were randomly selected for a retest via immunofluorescence detection of viral IE antigens, but none of them had an effect on focal growth as compared to plasma from an HCMV-seronegative donor. Bars represent mean values of 10 foci, error bars represent the SEM. (**C**) Representative images from three of these seven retested plasmas are shown.

The mean relative luciferase activity of the antiviral controls was around 0.12 (=12%), reflecting maximal inhibition of focal growth. None of the plasma samples reached this value, indicating that none of them could substantially block cell-to-cell spread of the Merlin strain (Figure 2A). Nevertheless, seven out of the 14 top scorers in the luciferase screening were randomly selected (representing relative luciferase activities between 0.22 and 0.50) and re-evaluated for cell-to-cell spread inhibition in an immunofluorescence-based assay that visualized viral IE antigen in the nuclei of infected cells and thereby allowed to assess focus size at 6 d p.i.. None of these seven plasma samples notably reduced focal growth of strain Merlin when compared to HCMV-seronegative control plasma, whereas formation of foci was completely blocked by direct-acting antivirals (Figure 2B,C). Obviously, the few hits that had at least a partial effect in the screening assay represented statistical outliers rather than true biological activity against the cell-associated spread of HCMV.

### 3.3. Cell-Type-Dependent Differences in the Degree of Resistance against Neutralizing Sera

For our screening approach, we used plasma samples from the same donor population that we had previously tested regarding the neutralization of a cell-free virus [37]. It was remarkable that none of the “elite” donors with high and broad neutralization capacities against cell-free HCMV strains showed a notable effect on cell-to-cell spread of our reporter virus. To exclude the possibility of strain-specific resistance of the Merlin derivative that we used in the screening approach, we tested six of these highly neutralizing serum samples in a targeted approach against three strictly cell-associated recent HCMV isolates. To assess whether our fluorescence-based focus expansion assay was able to detect inhibition of focal growth in principle, we also included endothelial cells. Cell-associated HCMV spread in epi- and endothelial cells has repeatedly been reported to be at least partially sensitive to inhibitory antibodies with some virus strains [31,32,34,35]; hence, we could expect partial effects of our highly neutralizing serum samples in these cell types.

**Figure 3:**
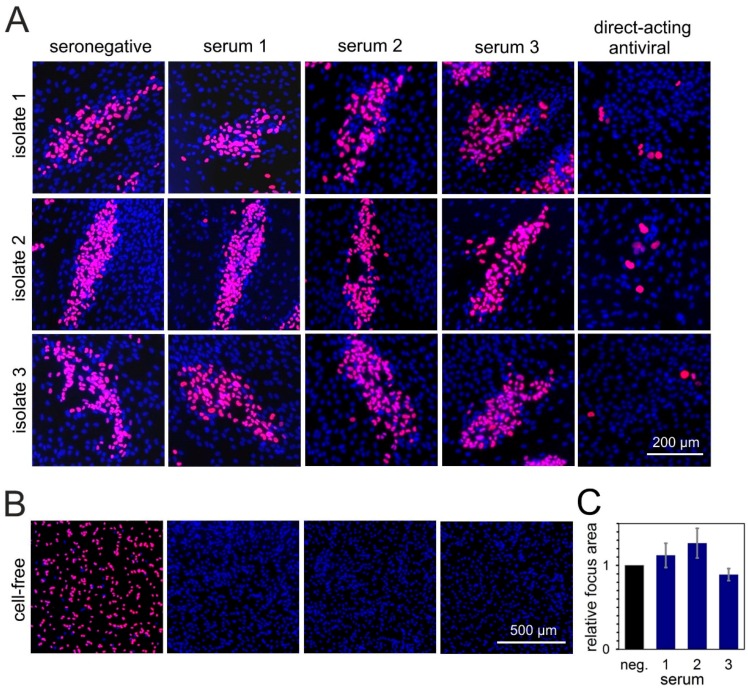
Sera with highly neutralizing capacity against cell-free HCMV transmission cannot inhibit cell-associated spread of clinical isolates in fibroblasts. (**A**) Fibroblasts infected with cell-associated clinical HCMV isolates were cocultured with an excess of uninfected HFFs, and, the following day, sera with highly neutralizing capacity against cell-free HCMV transmission were added to the coculture. Addition of seronegative human serum or a direct-acting antiviral served as a negative or positive control, respectively. Cocultures were then incubated for six days more to allow for cell-associated viral spread, followed by fluorescence staining for viral IE antigens (red signal). Nuclei of cells were stained with DAPI (blue signals). Three representatives out of six tested sera are shown. (**B**) To prove their neutralizing capacity, sera were mixed with HCMV-TB40/E-BAC4 (MOI 1) and incubated for 2 h. Fibroblasts were then infected with the serum-virus mixture. The next day, IE antigens were detected via indirect immunofluorescence staining (red signals). (**C**) For the three sera shown in panel A, focus areas were determined and compared with the negative control. For each serum, the bar represents the mean value of the three isolates (four to seven foci evaluated per isolate). Error bars represent the SEM.

HFFs infected with the clinical isolates were cocultured for 6 d with an excess of HFFs or HEC-LTTs in the presence of sera known to have a high neutralizing capacity. Focal growth was visualized by indirect immunofluorescence staining of viral immediate-early antigens, and the number of antigen-positive nuclei per focus was evaluated as a readout for spreading efficiency of the virus under the various conditions. Focus formation was almost completely inhibited by the direct-acting antiviral agent ganciclovir, which served as a positive control. In the presence of serum from an HCMV-seronegative individual, which served as a negative control for unimpeded spread, large foci containing around 100 infected cells formed in HFF cocultures. Compared with this negative control, none of the six highly neutralizing sera could notably reduce the focus size in HFFs (Figure 3A,C), while their neutralizing capacity was concurrently confirmed by a complete inhibition of the cell-free virus (Figure 3B).

**Figure 4:**
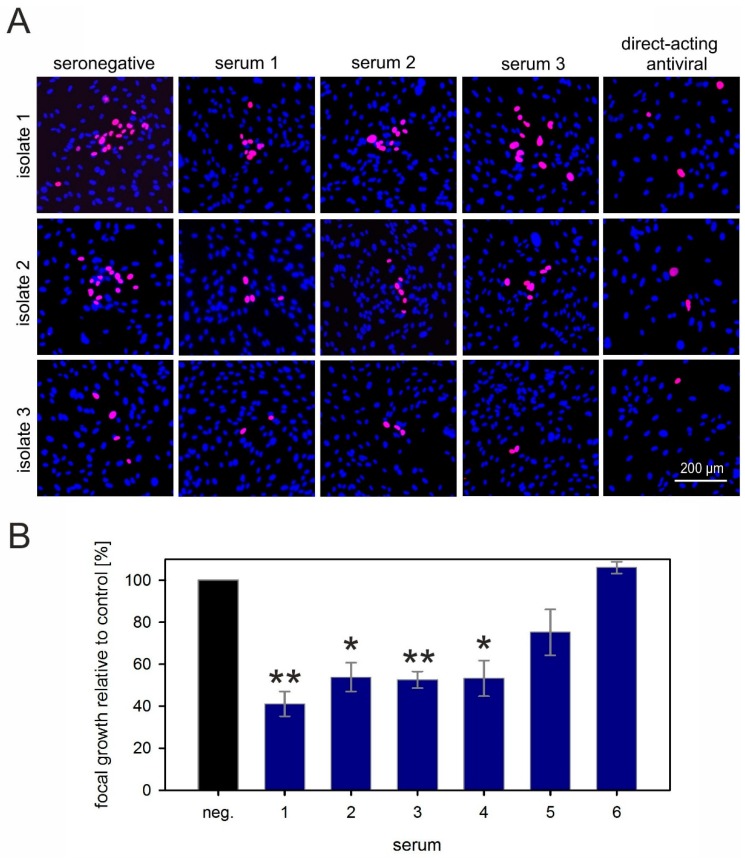
Highly neutralizing sera can partially reduce cell-associated spread of HCMV in endothelial cells. (**A**) Fibroblasts infected with cell-associated clinical HCMV isolates were cocultured with an excess of endothelial cells (HEC-LTTs) and, the following day, sera from highly neutralizing blood donors were added to the coculture. Addition of seronegative human serum and a direct-acting antiviral served as a negative and positive control, respectively. The cocultures were then incubated for six days more to allow for cell-associated viral spread, followed by fluorescence staining for viral IE antigens (red signal). Nuclei of cells were stained with DAPI (blue signals). Three representatives out of six tested sera are shown. (**B**) To quantify the effect of sera on focal growth, the overall numbers of infected cells per well were counted and divided by the number of infected cells cultured in HCMV-seronegative serum (neg). Each bar represents the mean value obtained with three different clinical isolates. Error bars reflect the SEM. Asterisks indicate significant (*) or highly significant (**) differences as compared with the control (neg).

In HEC-LTT cocultures, the isolates formed smaller foci of around 20, 14, or four infected cells in the presence of HCMV-seronegative serum. As expected, some of the highly neutralizing sera partially inhibited the focus expansion in this cell type. Depending on the serum and the isolate, focus size was reduced to a variable degree, with about 50% reduction being the maximal effect that we observed (Figure 4A). These sera reduced not only the focus size but also the number of foci. However, as the foci were often not very confined, we considered that counting the total number of infected cells may be more accurate to evaluate the overall inhibitory effect. Therefore, we determined the total number of infected cells per well, showing that four out of six highly neutralizing sera significantly inhibited growth of clinical isolates in endothelial cells by 50% to 60% (Figure 4B; *p* values between 0.005 and 0.02), whereas one serum completely failed in this cell type, and reduction by one serum was not significant. To exclude the possibility that the inhibitory effect on focal growth in endothelial cells was due to the release of free virus in this cell type, we tested supernatants of endothelial-cell cocultures, but never found indications of cell-free infectivity with any of the isolates.

In summary, even exceptionally effective neutralizing sera could not inhibit the cell-associated spread of HCMV in fibroblasts, while the majority had the expected partial effect on cell-associated spread in endothelial cells.

### 3.4. Neutralizing Serum Fails to Reduce Cell-To-Cell Transmission of HCMV Particles

A possible explanation for viral cell-to-cell spread, despite the presence of highly neutralizing anti-HCMV antibodies, might be provided by the assumption that numerous virions are transmitted during cell-associated spread, resembling infection at high MOIs [56]. Based on this consideration, we hypothesized that highly potent neutralizing antibodies may reduce the number of transferred particles, but may not be sufficient to prevent infection, which can be accomplished by a single virion that is not inhibited.

To enable investigation of cell-associated HCMV spread on a single-particle level, a dual-labeled HCMV was generated on the genetic background of HCMV clinical isolate-like strain Merlin. The envelope glycoprotein M (gM) was labeled by insertion of the red fluorescent protein mCherry directly after the first transmembrane region, and EGFP was fused to the C-terminus of the capsid-bound tegument protein pp150. When coculture experiments are performed with this dual-fluorescent mutant, virus particles that are transferred from a late-stage infected cell to neighboring cells can be detected and enveloped particles (red and green signal, yellow dot-like speckles) can be distinguished from naked viral capsids (green dot-like speckles). This virus was then employed to analyze the effect of neutralizing plasmas on the number of virions transferred during cell-to-cell spread. HFFs were infected with the dual-labeled Merlin, at 1 d p.i. plasma was added, and, at 5 d p.i., cells were fixed, and the red fluorescent signal was enhanced via indirect immunofluorescence for easier evaluation. Using this approach, we tested nine plasma samples with previously identified potent neutralizing capacity [37] and compared them with plasma from an HCMV-seronegative individual as a negative control.

**Figure 5:**
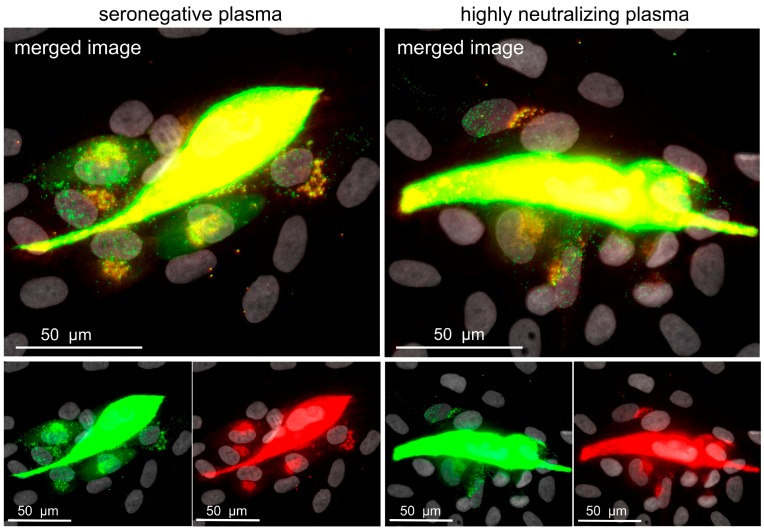
Highly neutralizing plasma does not reduce the number of transferred viral particles. Fibroblasts were infected with the dual-labeled Merlin-pAL1502-UL100mCherry-UL32EGFP, and treated either with plasma with highly neutralizing capacity against cell-free HCMV transmission or with HCMV-seronegative plasma on the following day. Five days after infection, cells were fixed, mCherry signals were enhanced with indirect immunofluorescence, and cultures were investigated with a fluorescence microscope. A representative image of each condition is shown. The viral capsid-associated tegument protein pUL32 is visualized in the green channel, whereas viral envelope protein gM is visualized in the red channel. Dot-like dual-fluorescent signals appear yellow in the merged image and represent enveloped viral particles, whereas naked viral capsids are represented by green-only dot-like signals. Nuclei were counterstained with DAPI (grey).

First of all, our findings with the seronegative plasma supported the assumption of high infection multiplicity during cell-to-cell transmission of HCMV. Between 10 and more than 50 virions were found on cells in the surroundings of late-stage infected cells, and the majority of these virus particles accumulated at the nuclei of the respective cells. Most of them did not contain an envelope signal, as indicated by the fact that they only had a green signal but lacked red fluorescence (Figure 5).

Few virus particles showed red and green signals, suggesting that complete virion particles were released by the productively infected central cell, and that such particles lost their envelope during penetration into the neighboring cell. This interpretation was further corroborated by the accumulation of red fluorescent gM signals at a perinuclear site in cells where many green particles were detected at the nucleus. Regarding a possible effect of neutralizing plasmas, we could not observe differences in the number of transferred viral particles when the nine highly neutralizing plasma samples were compared with the negative control. This led us to reject our hypothesis that the resistance of cell-to-cell spread against neutralizing plasmas is due to a greatly reduced but still-sufficient particle transfer in the presence of such plasma. Obviously, cell-associated virus transfer is completely unaffected by anti-HCMV antibodies contained in such plasma samples.

## 4. Discussion

Directly after isolation from clinical specimens, HCMV grows focally in fibroblast cultures without detectable cell-free infectivity in the supernatant [26,27]. It is, therefore, tempting to speculate that in vivo HCMV also can spread in a cell-associated fashion, something that is supported by the lack of detection of cell-free virions in plasma, as well as the ability of leucodepletion to prevent virus transfer [39,40,41]. During adaptation to cell culture, viral gene loci RL13 and UL128L are modified, infectious progeny becomes detectable in the supernatant, and the virus can now spread both in a cell-associated and a cell-free fashion [28,29,36]. Cell-free and cell-associated spread are usually distinguished by the inhibitory effect of neutralizing antibodies: the antibody-sensitive part of the infectivity is regarded “cell-free”, whereas the antibody-resistant part is regarded as “cell-associated” [57]. Fitting with this definition, recent HCMV isolates that do not release detectable infectivity into the supernatant (as well as isolates engineered to retain the cell-associated phenotype of clinical isolates) are almost completely resistant to neutralizing antibodies [30,36,58], suggesting a fundamental mechanistic difference between the two transmission modes.

The disappointing results with the clinical application of antibodies for the treatment of HCMV infection may at least in part be explained by the assumption that HCMV can spread in a cell-associated fashion in vivo, and that this mode of transmission cannot be prevented by anti-HCMV immunoglobulin preparations. In line with that, the partial protection elicited by a recombinant gB vaccine is obviously not mediated by increased capacity of antibodies to inhibit cell-associated spread [23]. This is, to some extent, surprising because the core fusion machinery of HCMV, which is targeted by neutralizing antibodies, is not only required for cell-free but also for cell-associated spread. This can be concluded from the fact that mutants lacking gB, gH, and gL cannot be reconstituted at all, whereas mutants lacking gO can form small plaques because only cell-free infectivity is specifically affected [59,60,61]. It is, therefore, rational to propose that, in principle, viral fusion machinery can also be targeted by inhibitory antibodies to prevent cell-to-cell spread. Resistance of recent clinical isolates against currently available anti-HCMV immunoglobulins, however, indicates that these preparations have either too little potency or the wrong specificity to be effective against cell-associated focal spread.

Previous work showed that activity against cell-associated spread is not common in sera from HCMV-seropositive individuals or pooled preparations like hyperimmunoglobulins [30,36,58], which did, however, not exclude the possibility that a few individuals might have exceptionally potent antibodies in their plasma that can inhibit the cell-associated spread of HCMV. To test this possibility, we aimed to apply a high throughput approach for detection of cell-to-cell inhibitory antibodies to a large blood-donor population that we had recently analyzed for neutralizing capacities against a cell-free virus [37]. Therefore, a Gaussia luciferase-secreting reporter virus was generated on the genetic background of a variant of the Merlin strain that contains intact RL13 and UL128L regions and, therefore, spreads in a cell-associated fashion like recent clinical isolates [28]. Luciferase activity in the supernatant of infected cultures was shown to reflect cell-associated focal growth of the virus, and hence was a valid readout to screen for donors that can reduce this mode of transmission by their plasma.

Remarkably, none of the 8400 plasma samples that we screened could block the focal spread of the Merlin reporter virus to a similar extent as the direct-acting antiviral agents that we used as controls. Furthermore, when the samples with the lowest luciferase values in the screening were retested and additionally evaluated in an independent, more accurate fluorescence-based assay, not even a partial effect on viral spread could be reproduced, suggesting that their low values in the initial screening were due to test variation rather than a real antiviral effect. This surprisingly clear negative finding emphasizes how effectively HCMV escapes from antiviral antibody responses by its cell-associated transmission mode. To exclude the possibility that this is a strain-specific effect, we tested the inhibitory effect of six sera with high neutralizing capacity against cell-free HCMV on three recent cell-associated patient isolates and showed that none of the six sera could inhibit cell-associated spread of these isolates in fibroblasts at concentrations that completely blocked cell-free transmission of strain TB40/E. Concerning our initial working hypothesis, this means that, if donors with cell-to-cell-inhibiting plasma exist at all, they are extremely rare, a result that is consistent with the inability of pooled antibodies from HCMV-seropositive donors to inhibit cell-associated transfer into multiple cell types [36].

This clear failure of anti-HCMV sera regarding inhibition of a cell-associated virus spread is hard to reconcile with the notion that cell-to-cell spread depends on the same glycoproteins that are necessary for cell-free infection. One possible explanation could be that cell-associated spread occurs at high infection multiplicity [56]. If numerous virus particles were transmitted from productively infected cells to their neighboring cells in a growing focus, and antibodies neutralized the vast majority of such particles, a single successful particle would still be sufficient to initiate the replication cycle in the neighboring cell. In this scenario, the reason for antibody resistance would be a quantitative rather than a qualitative issue. To address this, we generated a dual-fluorescent mutant of the cell-associated Merlin strain, in which viral envelope protein gM was tagged with red fluorescent protein mCherry and the capsid-associated tegument protein pp150 was tagged with green fluorescent protein EGFP [62]. As expected, infection multiplicity in a growing focus was high, with several dozens of virions being transferred from the productively infected cells to their neighboring cells. However, the presence of highly neutralizing antibodies did not reduce the number of virus particles. Given that the HCMV fusion machinery is necessary for cell-to-cell transmission of viral progeny, the simplest explanation is that the sites where neighboring cells interact during transmission are not accessible to antibodies, leaving the possibility that smaller fusion inhibitors might be more successful, e.g., small peptides derived from PDGFR-alpha [63] or small compound drugs that can interfere with the function of viral glycoproteins. Our test system can now also be applied to screen such compounds for their cell-to-cell spread-inhibiting activity.

To some extent, antibodies with highly neutralizing capacity against cell-free HCMV infection could inhibit cell-associated viral spread in endothelial cells, whereas they completely failed in fibroblasts. This is in agreement with previous reports that pentamer-dependent cell-to-cell spread (in epi- and endothelial cells) is more sensitive to anti-HCMV antibodies [31,32,34,35]. Inhibition was, however, never complete, even with the most potent neutralizing anti-HCMV sera. Assuming that partial resistance of pentamer-dependent spread and complete insensitivity of pentamer-independent spread also applies to the situation in vivo, strategies for inhibition of the cell-associated spread of HCMV are desired, as they might provide a basis to improve the clinical efficacy of anti-HCMV treatment regimens. Of course, direct-acting antivirals serve this purpose. However, in situations where their use is limited by adverse effects or the occurrence of resistant strains [11,12], alternatives with a different mode of action may be required.

It is important to mention that our results do not absolutely exclude the possibility that antibodies might be effective against cell-associated spread in fibroblasts, but they indicate that such antibodies cannot be detected easily in donor plasma or serum. Still, a possibility remains that certain antibody specificities exist that are underrepresented in whole serum and, therefore, not concentrated enough to exert their effect in our screening assay. To search for such specificities, cloning of complementarity-determining regions from individual B cells [64], followed by screening with the Gaussia luciferase reporter virus, might be an option. These results also do not argue against a contribution of the humoral immune response in general for the control of HCMV. It is conceivable that neutralizing antibodies can be beneficial at certain steps of the pathogenesis, e.g., the initial transfer of the virus from host to host, and that more indirect effects of anti-HCMV antibodies may help control viral spread in interplay with other components of the immune system, as in antibody-dependent cellular cytotoxicity (ADCC).

## 5. Conclusions

Screening of a large blood-donor population with a Merlin-derived reporter virus could not identify individuals that can inhibit the cell-associated spread of HCMV by their plasma, underscoring the remarkable capability of HCMV to evade the host’s antibody response via this transmission mode. Future attempts to inhibit cell-to-cell transmission of HCMV particles may, therefore, need to focus on smaller molecules to facilitate access or on exceptional antibody specificities, which are usually underrepresented in donor plasma.

Successful treatment of HCMV spread should target both cell-free as well as cell-associated spread. In previous work, we demonstrated that a small subpopulation of blood donors carry antibodies with high neutralizing capacity in vitro that might also reduce cell-free spread in vivo. Since this study shows the limitations of an antibody-based approach regarding cell-associated spread, future studies should explore combinations of antibodies with high neutralizing capacity and other pharmacological interventions targeting focal spread.
